# The shape of the pill: Perceived effects, evoked bodily sensations and emotions

**DOI:** 10.1371/journal.pone.0238378

**Published:** 2020-09-08

**Authors:** Olesya Blazhenkova, Kivilcim Dogerlioglu-Demir

**Affiliations:** 1 Faculty of Arts and Social Sciences, Sabanci University, Istanbul, Turkey; 2 Sabanci Business School, Sabanci University, Istanbul, Turkey; Radboud Universiteit, NETHERLANDS

## Abstract

Current research examined the differential effects of pills’ shape (angular vs. curvy) on the perceived efficacy of the medicine, evoked bodily sensations and emotions. We investigated these effects by using different types of angular vs. curved stimuli: abstract drawn shapes (Study 1), 3D-printed mockup pills (Study 2) and photographs of the existing pills (Study 3). Participants were asked to imagine ‘taking’ angular and curved pills. They had to focus on the bodily sensations and report the evoked activations/deactivations in different body parts. Across three studies, we found that the angular pills evoke overall more activations in the body compared to curvy pills. We further reported differences in the topography of angular vs. curved pills’-triggered sensations in different body parts. Our results also revealed that angularity is linked with an energizing effect while roundness is associated with a calming effect. The shape effects were demonstrated not only in self-reported energized vs. calm subjective feelings but also in performance on a timed cognitive test. Compared to incongruent designs, pill designs (angular vs. curved) congruent with proposed drug benefits (energizing vs. calming) were perceived as more effective. Moreover, we found differences in emotions triggered by pills of different shapes. The present research provided new findings on angularity vs. curvature perception that may be valuable for cognitive psychology, marketing, pharmaceutical and supplements industry, and other applied fields.

## Introduction

In 2015, the US Food and Drug Administration approved the first 3D printed tablet: Spritam, an anti-epileptic seizure drug. Spritam has a unique structure, layered and porous that enables rapid dissolution [1, 2]. 3D printing of pills is obviously a breakthrough in pharmaceutical and dietary supplements industry as it creates many opportunities for custom designing tablets for every patient's characteristics (e.g., age, gender, medical history and even individual preferences) [3]. 3D printing also allows for precision since tablet shape determines drug release rates [4]. In effect, a comparison across five pill shapes (cube, pyramid, cylinder, sphere and torus) revealed that the fastest dissolution rates are obtained for pyramid shape formulations [5]. 3D printing clearly marks the future of pharmaceutical industry. Though most of the existing drugs have curved shapes (round or oval without sharp angles), as 3D printing becomes more widespread, we expect to see more diversity in pill shapes (e.g., angular with sharp corners), deviating from the norm [6].

**Fig 1:**
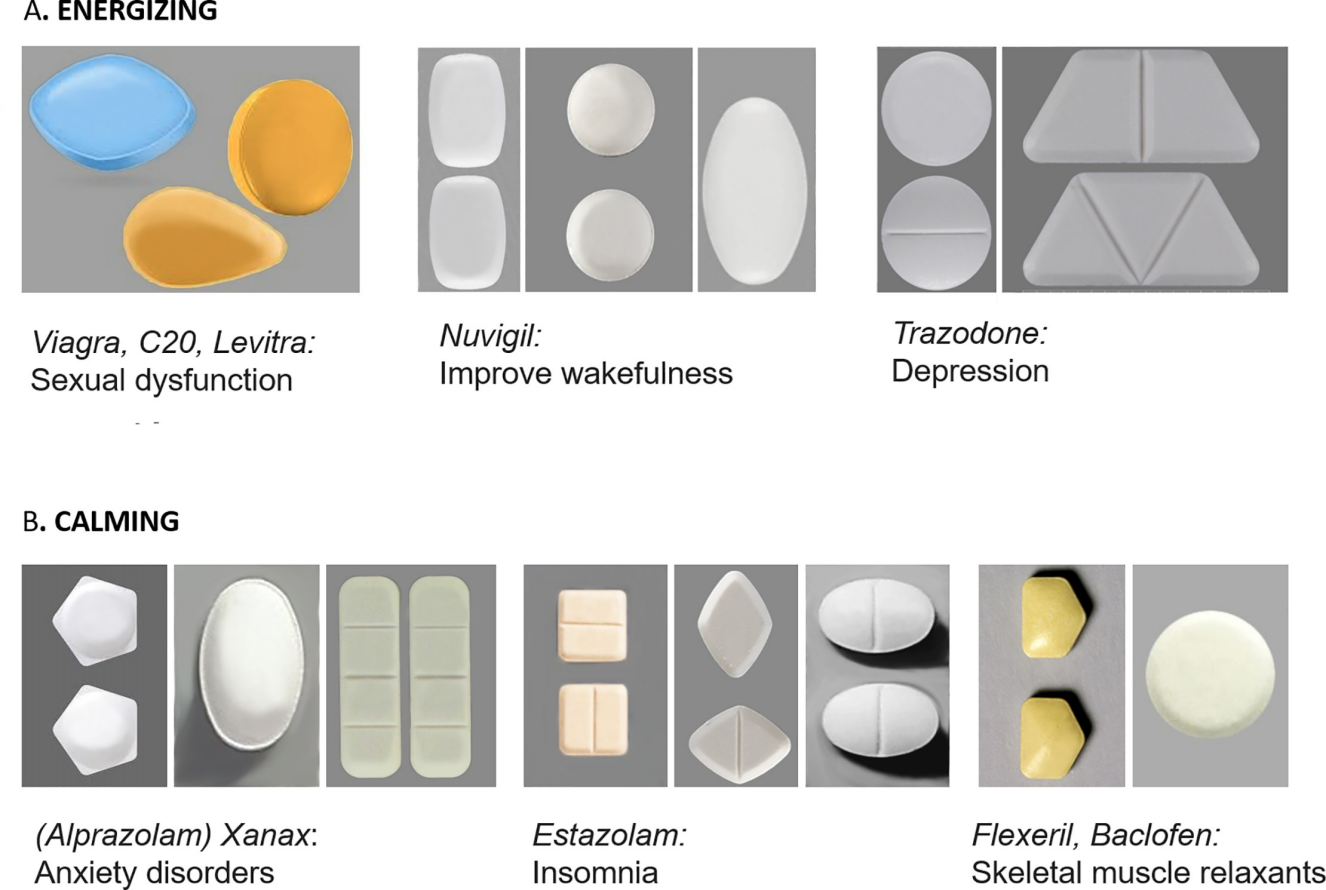
Examples of the existing angular vs. curvy pills.

While there is an increasing interest in new concepts in pill design, no research to our knowledge, has explored the relationship between the shape of the pills and the perceived benefit of the medicine. In this paper, we argue that when designing the pill, its shape (angular vs. curved) should also be taken into account as different shapes may be associated with different expectations about drug benefits: energizing (e.g., boosting energy, increasing alertness or wakefulness when tired or sleepy, enhancing focus and cognitive functions, boosting testosterone, stimulating athletes’ performance or treating sexual dysfunctions) vs. calming (e.g., treating anxiety, stress and sleep disorders, relieving pain or relaxing muscles). A review of the 2009 Physicians’ Desk Reference of medicine approved for sale in the USA reveals, however, that the choice of the shape of pills and supplements is rather arbitrary, irrelevant to the claimed benefit of the drug. For instance, ‘Energize’, an energy supplement that claims to improve focus and increase alertness, comes in round shapes. It is also possible to see both angular and curvy energizing pills, such as Viagra or Levitra that are used for men with erectile dysfunction by increasing blood flow to the penis (Fig 1A). Calming pills do not follow a shape pattern, either. Alprazolam is a sedative drug prescribed mainly for the treatment of anxiety, and its main benefits include relaxation and calming. Alprazolam tablets come in angular shapes (e.g., pentagon or rectangle) yet there are also curvy tablets available (Fig 1B).

Though the factors peripheral to the drug function are known to have an impact on patients’ expectations and actual efficacy of the medicine, the role of shape of pills seems to have been overlooked in pharmaceutical research. The role of extraneous attributes such as taste, size, color [7–9] and forms (e.g., injection, transdermal patch, capsule, tablet) [10–12] on patients’ expectations about the medicine have been consistently documented. For instance, it is known that consumers expect medicine with stronger side effects or bad taste to be more effective than those without such features [13]. Pink tablets are perceived as tasting sweeter than red tablets whereas yellow pills are perceived as tasting saltier [7]. While orange, yellow, and red pills are perceived as stimulating; green, blue, and purple pills are perceived as calming [7]. Such beliefs may lead patients to underuse or overuse prescribed medicine and potentially affect how much value they get out of it. As these expectations determine early and continued patient compliance [13], it is extremely important to take them into account in order to enhance efficacy perceptions in patients.

One of the factors that may contribute to efficacy perceptions is the perceived congruity resulting from a match between the characteristics of an item and expectations about the item. There is extensive research in marketing that demonstrates that while perceived congruity provides liking and satisfaction, perceived incongruity may lead to a sense of frustration and negative evaluations [14, 15–17]. That is, product design characteristics (shape, color and size) and brand design elements (logo shape, color and type font) influence consumer evaluations of products and their perceived benefits. For instance, judgments of the appropriateness of a font (e.g., typeface characteristics) of a logo for a product depend on the congruency between the connotative meaning of the font and the perceived brand personality of the product [16]. The logo font that is in line with the associated human characteristics of a brand leads to more positive consumer responses. In another study, compared with symmetrical logos, asymmetrical logos were discovered to be more arousing, leading to higher levels of excitement. As such, individuals perceive asymmetrical logos as more congruent with brands that have exciting personality [18].

Marketing literature is indeed replete with examples showing that consumers’ beliefs and expectations about the product influence user experiences. ‘Placebo effect of marketing actions’ refers to this exact phenomenon that marketing activities that are not inherent to the functionality of the product itself, such as pricing, packaging, logo and branding, can activate consumers' expectations about the product that may carry over to its actual efficacy [19]. For example, an energy drink may work much better (i.e., enhance consumers’ performance) when the price is high vs. low [20]. When it comes to shapes, marketing literature suggests that angularity vs. curvature may convey different meanings and benefits. For instance, angular brand logos denote conflict and aggressiveness whereas round logos are perceived as harmonious and gentle [21, 22]. As angularity depicts intensity, hardness and aggressiveness, an angular package (compared to a curvy one) leads consumers to experience the product taste as more intense [23].

Consistently, a great number of empirical studies of art and aesthetics, neurobiology, visual cognition and social psychology that reported differences in perception of angular vs. curved shapes [12, 24, 25–33]. Early research showed robust nonarbitrary associations between angular spiky versus curved cloud-like shapes and harsh- versus meaningless soft-sounding nonwords such as “takete” versus “maluma” [34] or “kiki” versus “bouba” [35]. These differences were suggested to be triggered by affective processes [24, 36–37] and underpinned by semantic and implicit associations [38]. For instance, Palumbo et al. [24] found that angular polygons were associated with words referring to danger and negative emotions, whereas curved polygons were associated with words referring to safety and positive emotions. Blazhenkova and Kumar [37] found consistently prevalent associations between abstract angular shapes with negative emotions and curved shapes with positive emotions. Different attributions for angularity vs. curvature in terms of emotional valence and arousal (e.g., unpleasant, agitating, and harsh vs. pleasant, gentle, quiet) were also documented in the literature [34, 39, 40].

Based on this literature, we expected that shape of the pill (angular vs. curved) will have an effect on the perceived benefit of the pill (energizing vs. calming), which may be enhanced when the shape is congruent with the suggested benefit. Furthermore, we hypothesized that angularity vs. curvature effects may manifest in differential sensations (activations vs. deactivations) throughout the body. This may be especially true for angular vs. curved pills’ effects on bodily sensations since pills are the agents that are supposed to be swallowed and to affect the body. Our expectation was based on the research demonstrating that angularity vs. curvature trigger different emotional associations [24, 34, 36, 37, 39, 40] as well as the work of Nummenmaa et al. [41] who revealed that different emotional states were consistently associated with topographically distinct subjective bodily sensations in terms of activations and deactivations. For example, happiness triggered activity across the entire body and sadness was associated with sensations of decreased limb activity. However, we do not have a specific hypothesis, but intended to explore and describe the pill-triggered activations and deactivations patterns in the body.

Herein we present three studies testing the effect of angular vs. curved shape of the pills on their perceived benefits (energizing vs. calming), the topography of shape-triggered bodily sensations, and the evoked emotions. We aim to test angularity vs. curvature shape effects by employing pill stimuli with different degrees of ecological validity: abstract black-and-white drawn schematics in Study 1, 3D-printed pills mockups in Study 2, and real pills’ photographs in Study 3. Across the studies, we tested the following hypotheses:

H1: The shape of the pill (angular vs. curvy) has energizing vs. calming effects, respectively (Study 1, Study 2, Study 3a, Study 3b).

H2: The proposed benefit of the pill (energizing vs. calming) leads to corresponding subjective effects (energizing vs. calming) (Study 3b).

H3: The effect (energizing vs. calming) is enhanced when the shape and the proposed benefit are congruent (energizing for angular and calming for curvy) and attenuated when they are incongruent (Study 3b).

H4: The angular vs. curved pills trigger different emotional associations (Study 3b).

## Study 1

This study examined the energizing vs. calming effects of shape angularity vs. curvature on the body by using drawn angular vs. curved abstract shapes (H1). In addition, we explored the topography of angularity- vs. curvature-triggered bodily sensations.

### Method

#### Participants

University students (*N* = 152) participated in the study. They were reimbursed with a course credit. The study was approved by Sabanci University Research Ethics Council (SUREC). Informed consent was obtained from all the participants via online software. Participants received the online link and completed the study online at their convenient time and location. This study was part of a larger study. To prevent random or inattentive answering, at the end of the survey, we asked participants to indicate whether they answered randomly (see details in the S1 File). The details of data cleaning procedures for this and the following studies are described in the S1 File. The data for this and the following studies was collected from all the available participants at the moment of data collection. The data from 147 participants (104 females, 18–25 years old, *M* age = 21.58) was retained.

#### Materials

**Fig 2:**
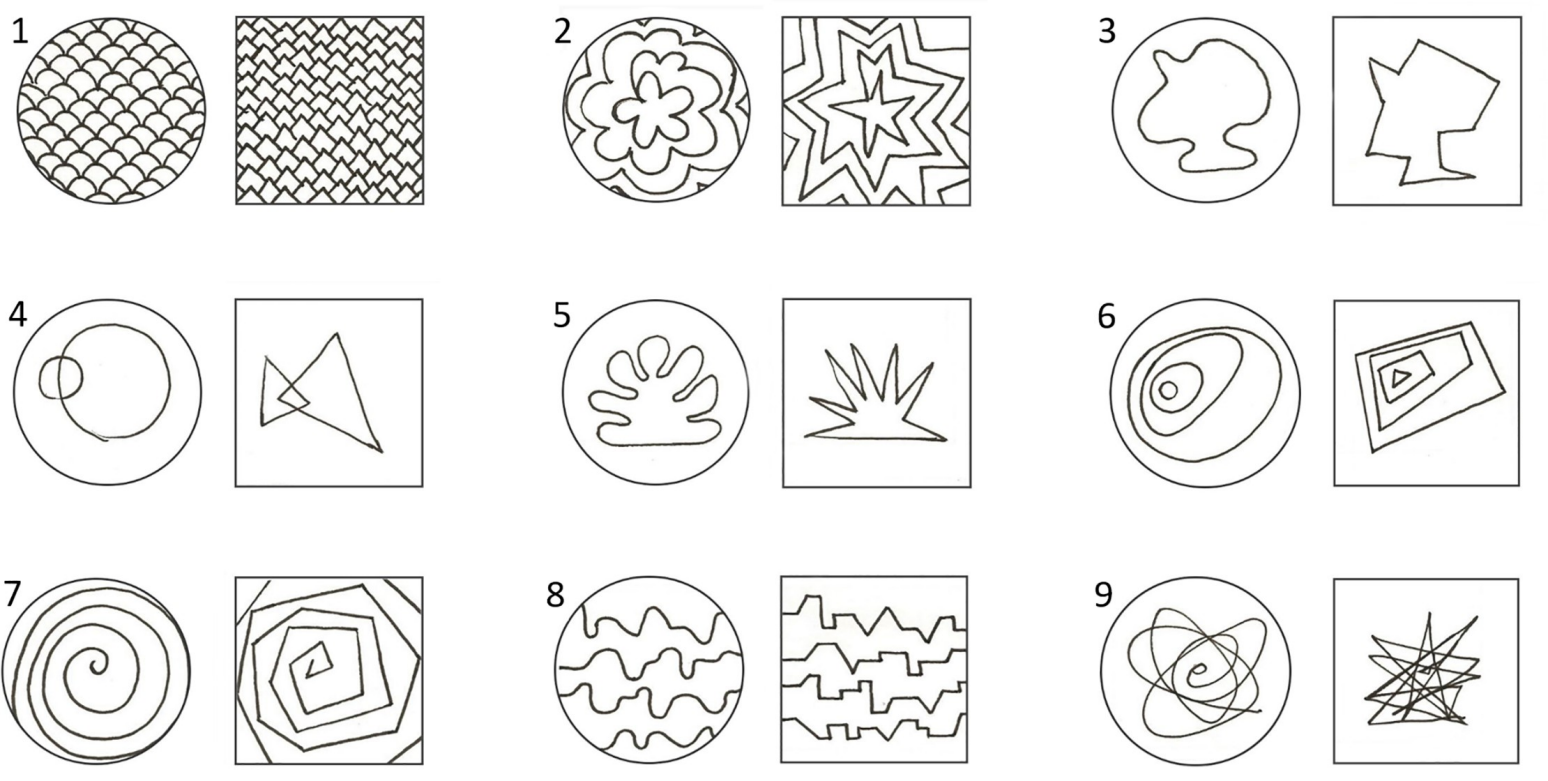
Angular vs. curved drawn pill stimuli used in Study 1.

Participants received the task that explored the perception of angularity vs. curvature through the associated bodily sensations. In the present task, participants were presented with 9 angular and 9 curvy abstract drawn shapes (Fig 2). These stimuli are freely shared at OSF [42]. To increase the resemblance with actual pills, angular shapes were presented in square frames while curved shapes were presented in round frames. The shape pairs varied in different characteristics, yet, within each pair, the shapes were drawn to maximally resemble each other, except for being angular or curved.

**Fig 3:**
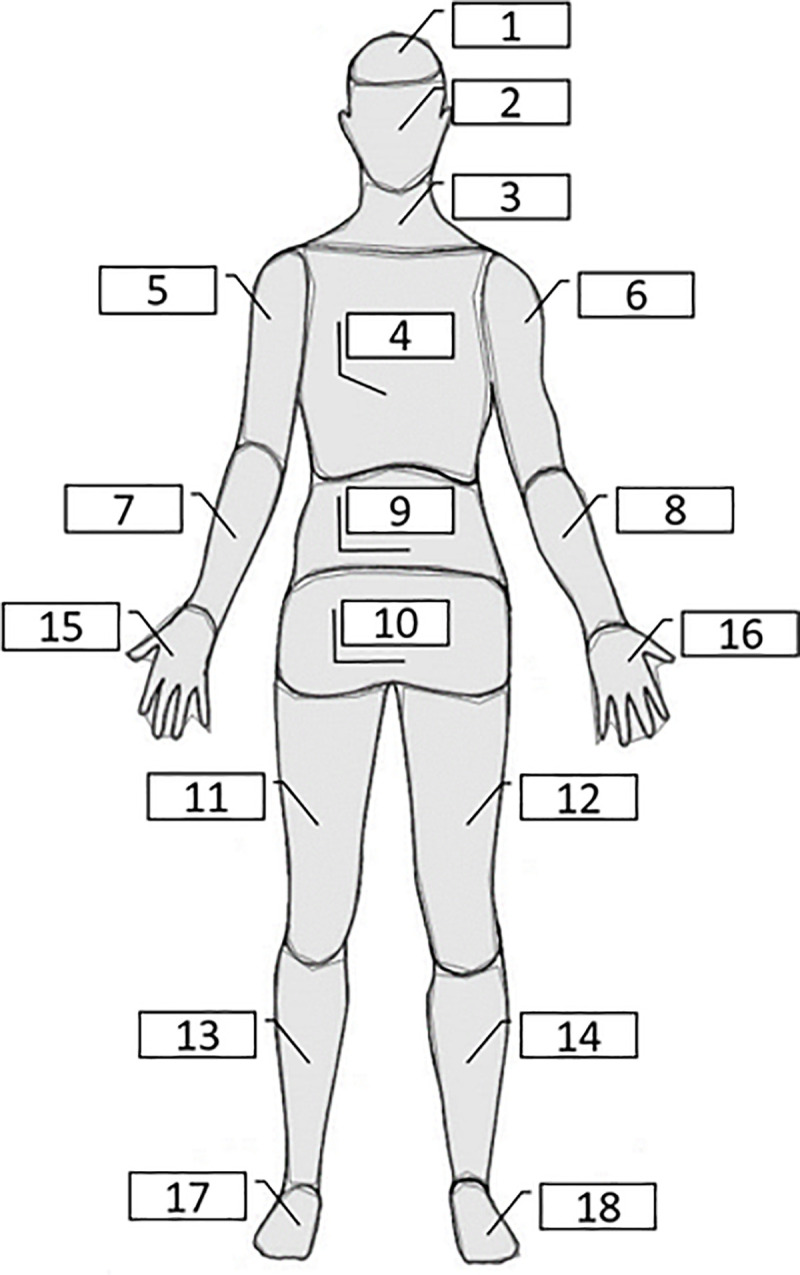
Body map used to indicate activations and deactivations.

Our task was created on the basis of the task developed by Nummenmaa et al. [41]. Similar to Nummenmaa et al. [41], participants were asked to indicate the evoked bodily sensations in terms of activations or deactivations on the body schema (Fig 3). The instruction was: “In this task, you will have to imagine that you are taking magic pills. They are completely safe and will affect your state for a very short period of time. You will see the drawings of these pills and then will have to imagine taking them. Please, focus on the sensations of your body. Try to locate your feelings inside your body. You may feel that activity in some parts of your body becomes stronger or faster, while, at the same time, the activity in other regions becomes weaker or slower”. To ensure that participants understood the instructions, they had to complete a practice trial in which they had to mark specific body regions. Without accurately passing this trial, they were not allowed to start the real task.

### Results

**Fig 4:**
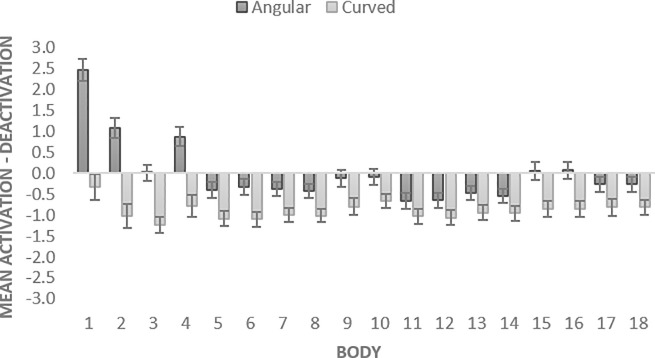
Activation-deactivation in bodily sensations evoked by angular vs. curved pills in Study 1. The bars represent SE.

For the convenience of the analysis, the activation-deactivation scores were computed as the number of activations minus the number of deactivations (e.g., positive score reflects that activations exceeded deactivations in a particular body area) separately for each body part. A 2 X 18 Repeated Measures ANOVA was conducted to assess the impact of two within-subjects variables, shape (angular, curved) and body part (1, 2, 3, 4, 5, 6, 7, 8, 9, 10, 11, 12, 13, 14, 15, 16, 17, 18, see Figs 4 and 12) on participants’ responses. The adjustment for multiple comparisons was done with Bonferroni correction (here and below, it was adjusted to the number of tests).

The analysis revealed a significant main effect of shape *F* (1, 146) = 58.649, *p* < .001, partial *η*^2^ = .287. The mean activation-deactivation values were significantly higher for angular than for curved shapes (*MD* = .906; *SE* = .118), *p* < .001. An additional one-sample t-tests with mean activation-deactivation values for angular and mean curved shapes (test value = 0) demonstrated that energizing effect of angular pills was not significant (*p* = .993), whereas calming effect of curved pills was significant (*p* < .001). The effect of body part was also significant, *F* (5.762, 841.319) = 13.406, *p* < .001, partial *η*^2^ = .084, with Greenhouse-Geisser correction. There was a significant interaction between shape and body part, *F* (6.310, 921.218) = 12.173, *p* < .001, partial *η*^2^ = .077, with Greenhouse-Geisser correction (Fig 4), (see S1 File for details).

### Discussion

Results of the Study 1 demonstrated that angular shapes evoked overall more bodily activations than rounded pill shapes (though, mostly due to the calming effect of curvature). Thus, our hypothesis that pills’ angularity evokes more activation than curvature on a body level (H1) was supported. This main effect of shape was qualified by a shape X body part interaction. Angular shapes evoked more activations especially in the head and chest areas. For curved shapes, deactivations were evident for all body parts except for the top of the head. Notably, the effect size was quite large for the shape effect but intermediate for body part effect and their interaction. In Study 1, we found differences in shape-evoked bodily sensations using simple schematic drawings of the pills that were not very realistic. To test whether the found effects would generalize to the perception of real-looking pills, we used realistic 3D mockups of the pills in Study 2.

## Study 2

Study 2, using the 3D-printed mockups of pills, further examined the shape effects on the evoked activations/deactivations of bodily sensations, as well as on the perceived benefits of the drugs using subjective self-report and performance measures (H1).

### Method

#### Participants

University students (*N* = 69, 48 females, 19–25 years old, *M* age = 21.58) participated in the study. They were reimbursed with a course credit. The study was approved by SUREC, and written informed consent was obtained from all the participants. Participants were tested individually in separate lab cubicles.

#### Materials

**Fig 5:**
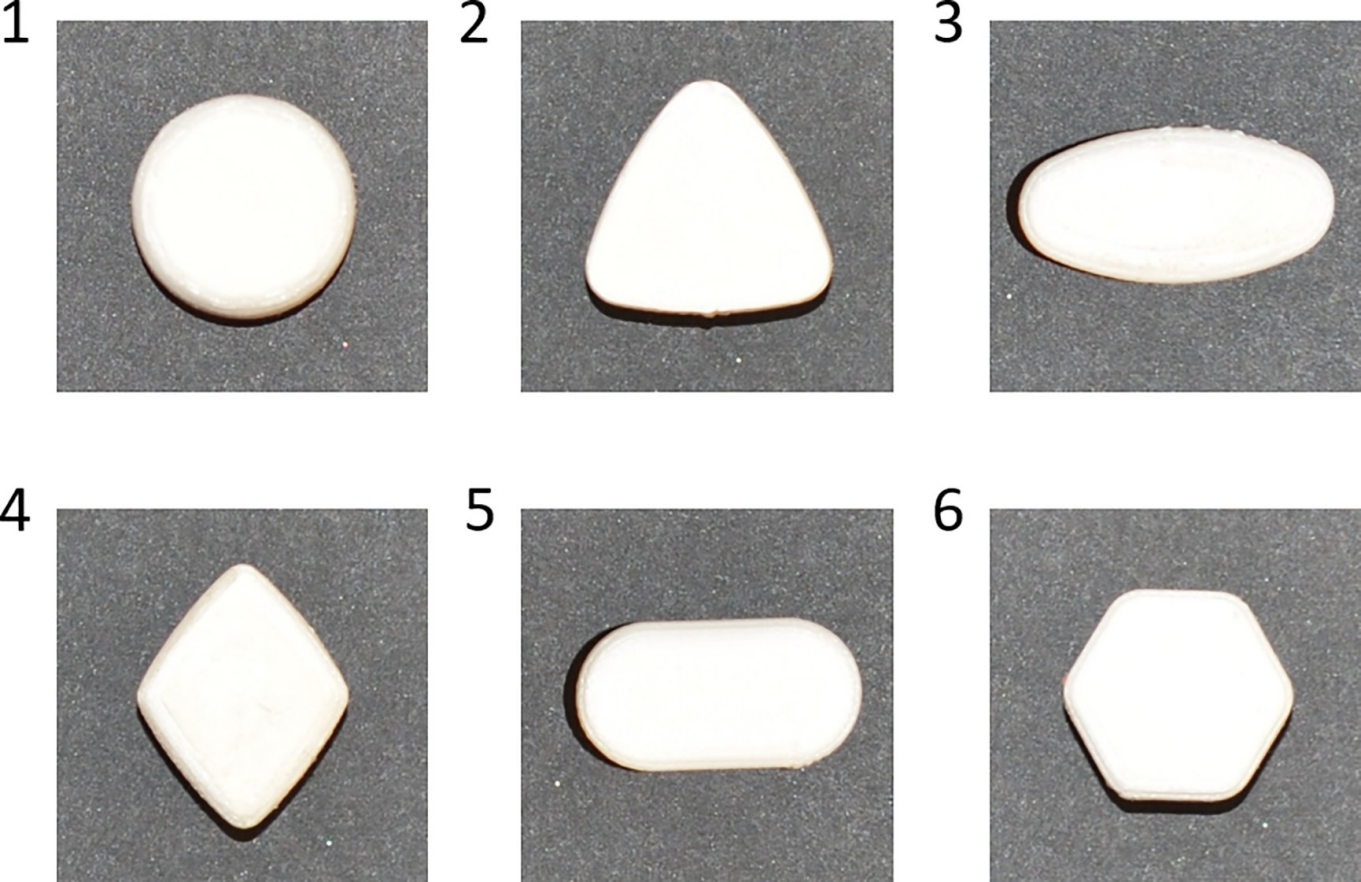
3D printed angular vs. curved pills’ stimuli used in Study 2.

Similar to Study 1, participants imagined ‘taking’ pills. This time they were given six 3D printed pills (one pill at a time). The participants were instructed in the following way: “In this study, imagine taking / swallowing these pills. These are completely reliable pills and will affect you only for a short time.” Then, participants were handed a pill, and were able to both visually inspect and haptically explore it. They were asked to pretend ‘swallowing’ the pill–make a real ‘swallow’ movement (without swallowing the actual pill). The order of administered angular and curved pill shapes was the same for all participants: 1. Curvy; 2. Angular; 3. Curvy; 4. Angular; 5. Curvy; 6. Angular (Fig 5).

After ‘taking’ each pill, participants responded to a paper-and-pencil survey, and answered the question about the current state feeling: *“*How do you feel after taking this pill?” using a 7-point scale (1 –Very Calm; 7 –Very Energetic). As a baseline, in the beginning of the study, participants answered the same question about the current state feeling “How do you feel now?”. For each pill, after rating the current state feeling, participants were instructed to “Imagine taking this pill. Focus on the feelings in your body and mark your feelings in the body chart below.” Participants had to use two separate body charts (Fig 3): 1) “Specify which fields are active (stronger or faster)?”, 2) “Which areas are weaker or slower?”.

Besides, participants completed Identical Pictures performance test [43]. This is a paper-and-pencil task which measures perceptual speed, the ability to quickly perform a simple visual perception task involving visual scanning and comparing figures. Participants were presented with arrays of five abstract black-and-white geometrical figures, and a target figure on the left. Their task was to quickly find and mark the figure in the array that is identical to the model figure (see the sample item in Supplement, S1 Fig in S1 File). Participants were asked to work as quickly as possible without sacrificing accuracy. Participants received 6 different subsets of Identical Pictures test each time after ‘taking’ one of the 6 pills, and then and completing self-report questions. One additional subset of this test was administered as a baseline at the beginning of the study prior to pill ‘taking’. Each subset consisted of 12 items and participants were given 17 seconds for completion. The test scores were computed as the number of objects marked correctly minus a fraction a point for each error (the proportion to the number of alternatives minus one for each item) to correct for guessing. The internal reliability (Cronbach’s alpha) of Identical Pictures test ranged from .81 to .84 [43], and it was .84 in the current study (the reliability was computed between 7 subsets of this test).

### Results

**Fig 6:**
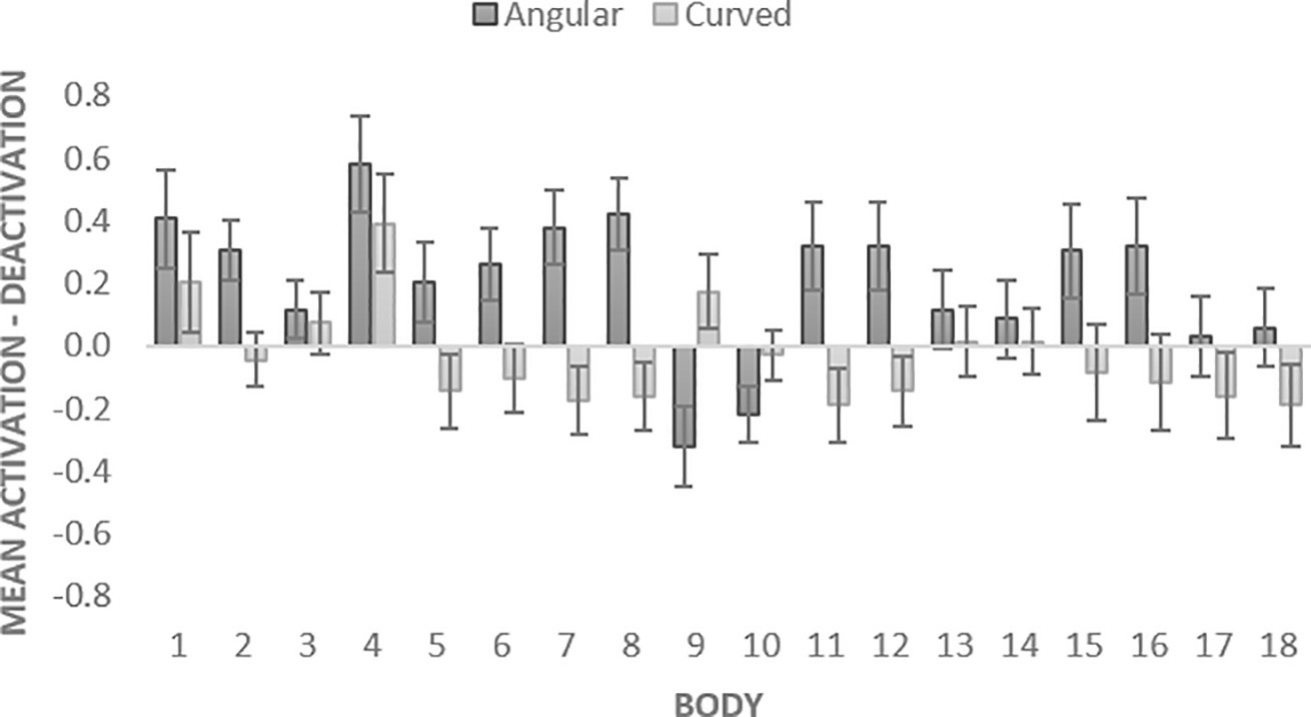
Activation-deactivation in bodily sensations evoked by angular vs. curved pills in Study 2. The bars represent SE.

To examine the topography of pill-triggered bodily sensations, same as in Study 1, the Activation-Deactivation scores were computed for each body area. A 2 X 18 Repeated Measures ANOVA (with Bonferroni correction) was conducted to assess the impact of two within-subjects variables, shape (angular, curved) and body part (1, 2, 3, 4, 5, 6, 7, 8, 9, 10, 11, 12, 13, 14, 15, 16, 17, 18, see Figs 6 and 12) on participants’ ratings.

The analysis revealed a significant main effect of shape *F* (1, 68) = 13.255, *p* = .001, partial *η*^2^ = .163. The mean activation-deactivation values were significantly higher for angular than for curved shapes (*MD* = -.242; *SE* = .066, *p* = .001). The additional one-sample t-tests with mean activation-deactivation values for angular and mean curved shapes (test value = 0) demonstrated that energizing effect of angular pills was significant (*p* < .001), whereas calming effect of curved pills was not (*p* = .361). The effect of body part was also significant, *F* (6.481, 440.714) = 2.290, *p* = .031, partial *η*^2^ = .033, with Greenhouse-Geisser correction. There was a significant interaction between shape and body part, *F* (7.853, 533.983) = 3.152, *p* = .002, partial *η*^2^ = .044, with Greenhouse-Geisser correction (Fig 6) (see S1 File for details).

**Fig 7:**
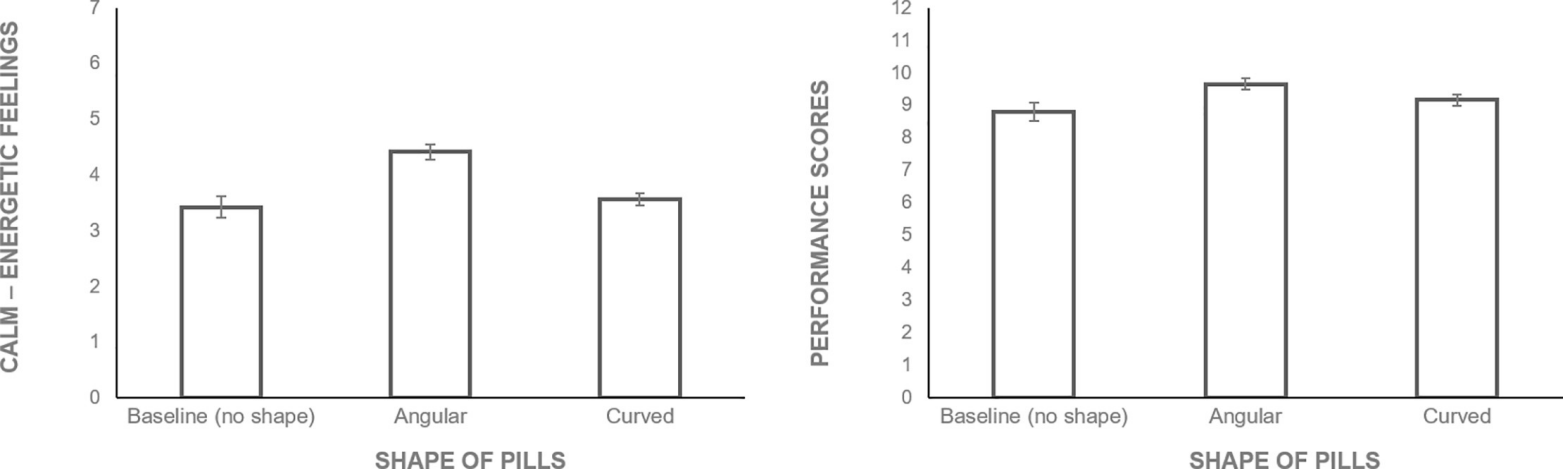
Shape-evoked differences in subjective and performance scores in Study 2. The bars represent SE.

To examine the subjective effects of pills, a 2 X 2 Repeated Measures ANOVA (with Bonferroni correction) was conducted to assess the impact of two within-subjects variables, shape (angular, curved) and time (before, after) ‘taking’ pills on participants’ ratings of their energetic-calm *feelings*. The analysis revealed a significant main effect of shape *F* (1, 68) = 25.205, *p* < .001, partial *η*^2^ = .270. The evoked energetic feelings were significantly higher for angular than for curved shapes (*MD* = .428; *SE* = .085, *p* < .001). The effect of time was also significant *F* (1, 68) = 21.855, *p* = .005, partial *η*^2^ = .112. There was a significant interaction between shape and time, *F* (1, 68) = 25.205, *p* < .001, partial *η*^2^ = .270 (Fig 7A) (see S1 File for details).

To examine pills’ effects on performance test scores, a 2 X 2 Repeated Measures ANOVA (with Bonferroni correction) was conducted to assess the impact of two within-subjects variables, shape (angular, curved) and time (before, after) on Identical Pictures Test [43] *performance*. The analysis revealed a significant main effect of shape *F* (1, 68) = 15.685, *p* < .001, partial *η*^2^ = .187. The performance was significantly higher for angular than for curved shapes (*MD* = .245; *SE* = .062, *p* < .001). The effect of time was also significant *F* (1, 68) = 6.959, *p* = .010, partial *η*^2^ = .093. There was a significant interaction between shape and time, *F* (1, 68) = 15.685, *p* < .001, partial *η*^2^ = .187 (Fig 7B) (see S1 File for details).

### Discussion

Consistently with Study 1, the results of the Study 2 demonstrated that angular shapes evoked overall more bodily activations than rounded shapes. Thus, our hypothesis that angularity will evoke more activation on a body level (H1) was supported with additional evidence, using realistic 3D pills. However, this difference was mostly due to energizing effect of angularity. As in Study 1, the main effect of shape was qualified by a shape X body part interaction. Angular shapes evoked more activations than deactivations, especially in the head, face, chest, and limbs (arms, hands and legs) areas, but evoked more deactivations in hip and abdomen areas. Unlike Study 1, curved shapes did not evoke significantly more deactivations than activations, and even yielded more activations in chest area. Furthermore, we found shape-evoked differences in the current state feelings ratings and performance test scores, qualified by a shape X time interaction. The found effects were consistent between self-report and performance test. Imagined ‘taking’ of angular but not curved pills led to more energetic subjective feelings as well as increased performance on a perceptual speed task. Curved pills did not evoke significant changes in subjective feelings or performance, as compared to the baseline. Both the data from pills-triggered bodily sensations, self-reported effects of pills, and results of the performance task suggest that the energizing effects of angular pills are more pronounced than calming effects of curved pills. Notably, for all the tasks the effect size of the shape was the largest compared to other effects and interactions. In order to further examine the effect of pill’s shape and its possible interaction with the proposed benefit of the pill, we conducted an extended Study 3 that involved a relatively large number of participants and explicit instructions manipulating the proposed energizing vs. calming benefits of the pills.

## Study 3a

The goal of Study 3a was to check whether the angular vs. curved pills stimuli, intended for use in study 3b, are indeed perceived as angular vs. curved. In addition, we examined whether angularity is more associated with an energizing effect, while curvature is associated with a calming effect (H1).

### Method

#### Participants

One hundred and twenty-two North American participants were recruited via Amazon Mechanical Turk (MTurk). Participants were tested used via an online software. The study was approved by SUREC, and informed consent was obtained from all the participants. They were reimbursed with monetary compensation. Prior to the analysis, the data cleaning was performed based on the attention check (see S1 File). The data from 118 participants was retained.

#### Materials

**Fig 8:**
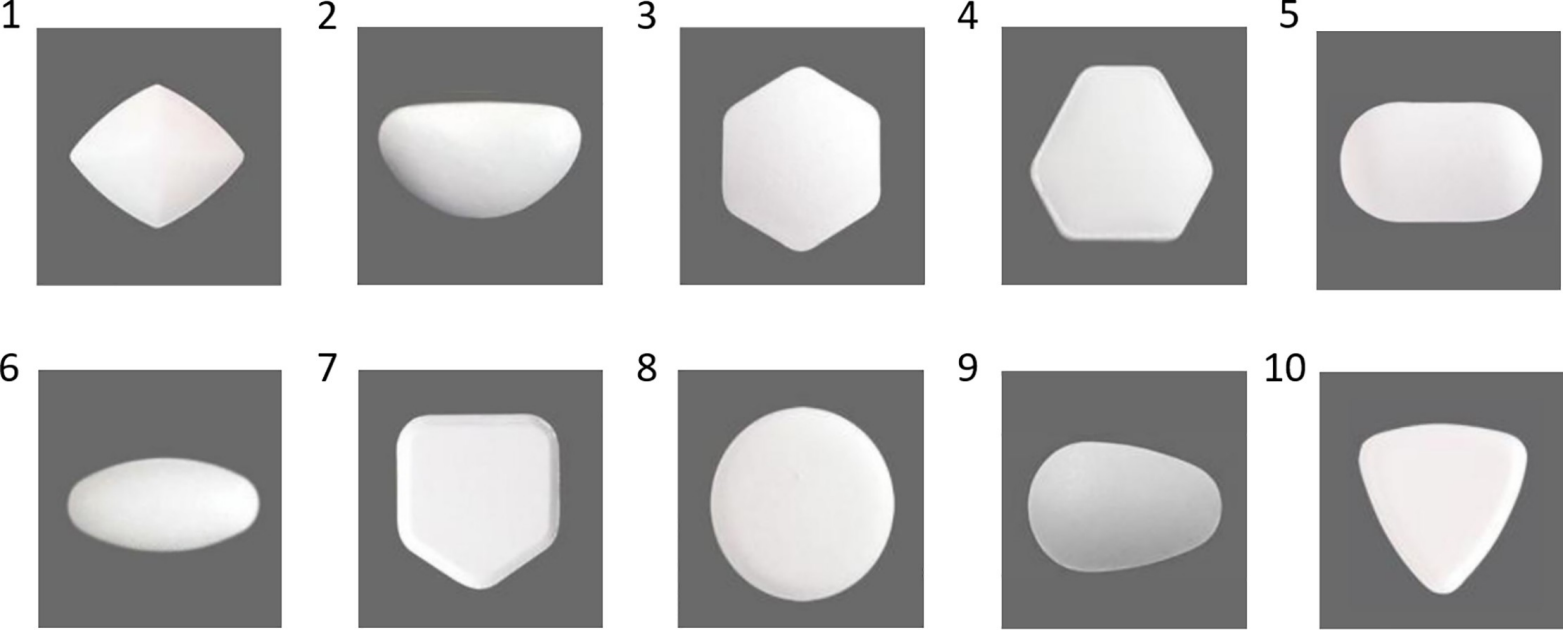
Angular vs. curved photograph pill stimuli used in Study 3.

Participants were presented with 5 angular (#1, 3, 4, 7, 10) and 5 curved (#2, 5, 6, 8, 9) shapes (Fig 8). We used white pills as they are perceived neutral [44]. Participants were asked two questions about the perceived shape of the pills: 1) “Please estimate the degree to which the figure above can be characterized as…” and gave answers on a 7-point scale (1—Angular; 7—Curvy); 2) “Please estimate the degree to which the figure above can be characterized as” and gave answers on a 7-point scale (1—Round-edged; 7—Sharp-edged). They were also asked about the effect of the pills: “Can you guess, what is the effect of this pill?” and gave answers on a 7-point scale (1—Calming; 7—Energizing).

### Results

Using paired-samples t-tests, we found that ‘angular–curvy’ mean ratings Curved pills were significantly higher than mean rating for angular pills (*MD* = 3.42, *SD* = 1.66), *t* (117) = -22.399, *p* < .001. Mean roundness-sharpness ratings for angular pills were significantly higher than those for Curved pills (*MD* = 2.74, *SD* = 1.29), *t* (117) = 23.116, *p* < .001. Mean Energizing effect ratings for angular pills were significantly higher than mean Energizing effect ratings for Curved pills (*MD* = 1.44, *SD* = 1.62), *t* (117) = 9.683, *p* < .001.

## Study 3b

The first goal of Study 3b was to replicate the pills’ shape (angular vs. curvy) effect on the perceived benefit of the pill (energizing vs. calming, respectively) (H1). However, while in Study 3a, the participants were directly asked about the potential benefit of the pill; Study 3b manipulated the instruction indicating the proposed benefit (calming vs. energizing). In addition, we included a neutral condition in which no benefit was stated as in studies 1 and 2. Study 3b examined whether the proposed benefit of the pill will lead to corresponding subjective effects (H2) and further explored the congruity effect of the suggested benefit and the shape of the pill on the effectiveness of the pill (H3). Study 3b also aimed to further investigate the effects of curvature and angularity on bodily sensations (H1) and emotional associations (H4).

### Method

#### Participants

University students (*N* = 548) participated in the study. They were reimbursed with a course credit. Participants were tested used via an online software. The study was approved by SUREC, and informed consent was obtained from all the participants via online software. They received the online link and completed the study at their convenient time and location.

Since, Study 3b was quite long compared to Studies 1, 2, and 3a, and it included multiple measures we expected that participants may experience fatigue and loss of motivation. Thus, to prevent random or inattentive answering due to the length of the study, we included two attention checks in the experiment and a question about possible random responding (see details of data cleaning in S1 File). The data from 418 participants (258 females; *M* age = 21.62, *SD* = 1.32) was retained.

#### Materials

Participants viewed photos of ten real pills (Fig 8). They were asked to indicate the evoked feelings, bodily sensations, and emotional associations. As in Studies 1 and 2, participants were instructed to imagine that they were ‘taking’ (swallowing) these pills. To examine the subjective effects of pills, we asked participants to indicate their feelings (1-very calm to 7-very energized) evoked by each pill. Then, to examine the topography of pill-triggered bodily sensations, participants were asked to focus on the sensations of their body and indicate them on the body schema. In the current study, in addition to the neutral condition as in Studies 1 and 2, we introduced two new conditions: energizing and calming. Participants were randomly assigned to one of the three conditions. The pills and the order of the presentation was the same across conditions except for the benefit stated (energizing, calming or not stated). After indicating the bodily sensations, participants were asked to view each pill again and to identify the emotional associations. We used a wheel format with answer options adopted from Geneva Emotion Recognition Test [45] depicting 14 emotions. Participants were allowed to select several answers for each shape.

### Results

#### Manipulation checks

To check whether the pills’ stimuli shapes were indeed perceived as angular vs. curved, the participants were asked to categorize all the 10 shapes into curvy vs. angular categories. Binomial tests (test proportion  =  .50) for all the pills demonstrated that angular pills were consistently identified as angular (pill 1–98%; pill 3–96%; pill 4–97%; pill 7–94%; pill 10–85%), whereas curved pills were consistently identified as curved (pill 2–99%; pill 5–99%; pill 6–99%; pill 8–99%; pill 9–99%), all *p’*s < .001.

#### Subjective energizing vs. calming effects of angular vs. curved pills

A 2 X 3 Mixed ANOVA (with Bonferroni correction) was conducted to assess the impact of shape (angular vs. curved) within-subject and between-subject benefit (energizing vs. calming vs. not-stated) variables on participants’ feelings rated on a 7-point scale (1 –Very Calm; 7 –Very Energized).

The analysis revealed significant effect of shape, *F* (1, 415) = 109.615, *p* < .001, partial *η*^2^ = .209, showing that pills’ angularity evoked more energizing feelings than curvature (*MD* = .554; *SE* = .053). An additional one-sample t-tests (test value = 4) demonstrated that both energizing effect of angular pills and calming effect of curved pills were significant (*p’s* < .001).

**Fig 9:**
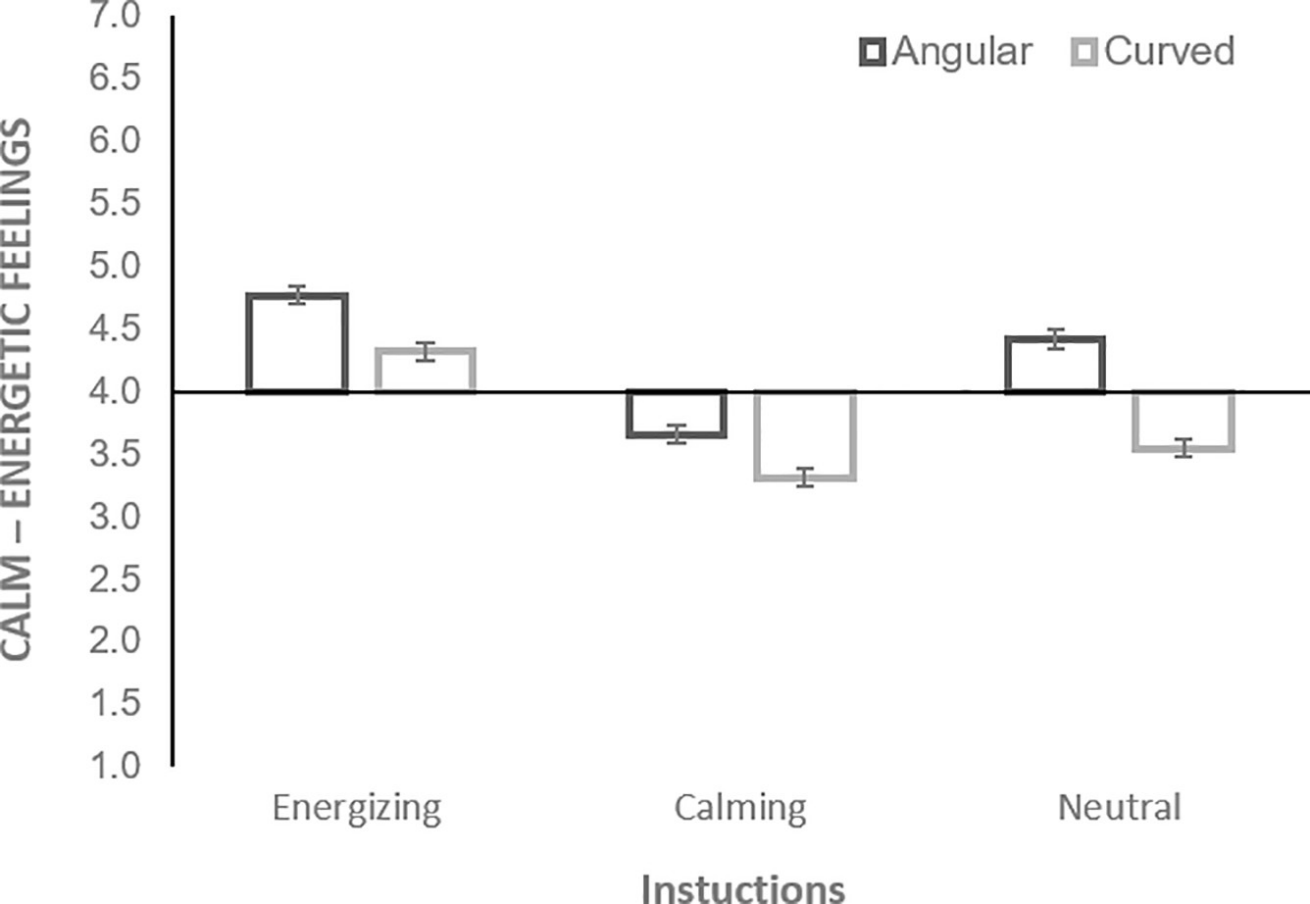
Energizing vs. calming effects of angular vs. curved pills in Study 3b. The bars represent SE.

There was a significant effect of benefit, *F* (2, 415) = 84.677, *p* < .001, partial *η*^*2*^ = .290, showing that Energizing instruction led to higher energized feelings than Neutral instruction (*MD* = .564; *SE* = .081) and Calming instruction (*MD* = 1.060; *SE* = .082), whereas Neutral instruction led to higher energized feeling than Calming instruction (*MD* = .496; *SE* = .080); all p’s < .001. There was a significant interaction between shape and benefit, *F* (2, 415) = 9. 573, *p* < .001, partial *η*^*2*^ = .044, suggesting that shape effect depended on the proposed benefit (Fig 9). The proposed energizing benefit yielded energizing effect for both angular and curved pills, however, this effect was greater for angular pills. The proposed calming benefit yielded calming effect for both angular and curved pills, however, this effect was greater for curved pills. Neutral instructions yielded energizing effect for angular pills, but calming effect for curved pills. Post hoc tests revealed significant differences between all the multiple comparisons (all p’s <001).

#### Body activation vs. deactivations effects of angular vs. curved pills

**Fig 10:**
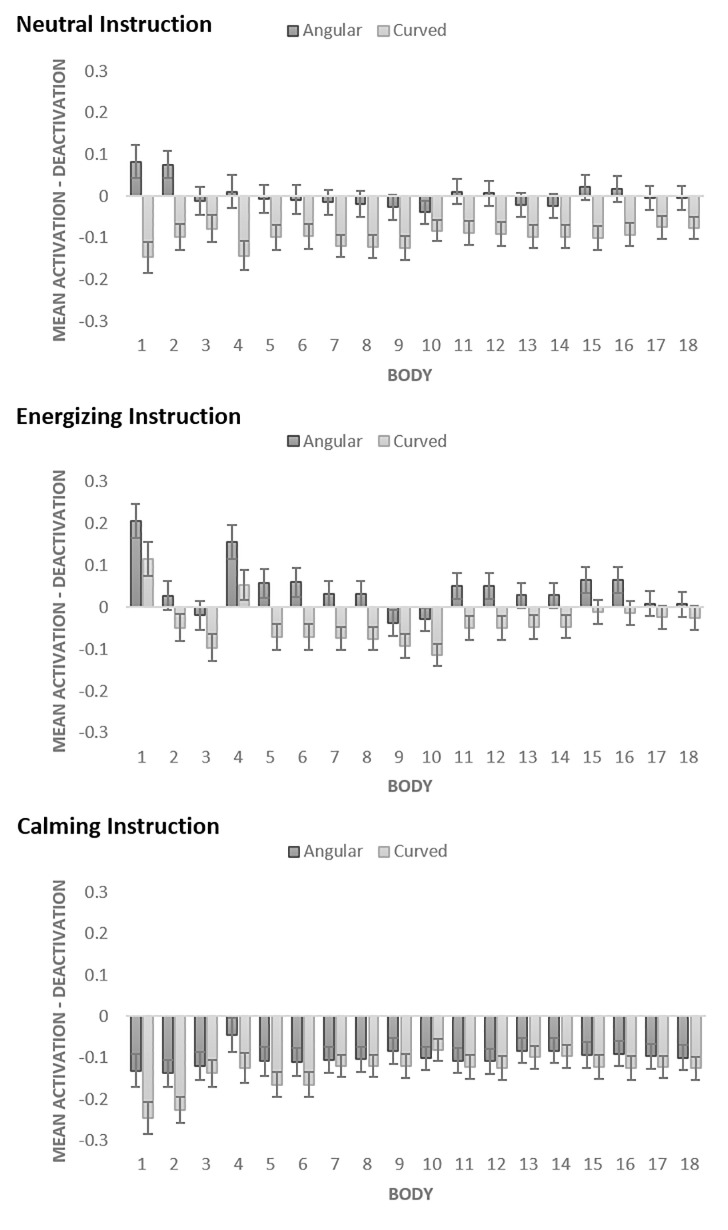
Activation-Deactivation in bodily sensations evoked by angular vs. curved pills in Study 3b. The bars represent SE.

Mixed ANOVA was conducted to assess the impact of within-subject shape (curved vs. angular), within-subject body part (1, 2, 3, 4, 5, 6, 7, 8, 9, 10, 11, 12, 13, 14, 15, 16, 17, 18), and between-subject benefit (energizing vs. calming vs. not-stated) variables on body activation-deactivation ratings (see Figs 10 and 12).

The results revealed a significant main effect of shape *F* (1, 415) = 40.875, *p* < .001, partial *η*^2^ = .090. The mean activation-deactivation values were significantly higher for angular than for curved (*MD* = .075; *SE* = .012) shapes (*p* < .001). The additional one-sample t-tests demonstrated that mean activation-deactivation for angular pills was not significantly different from zero (*MD* = -.018; *SD* = .26, *p* = .167), whereas calming effect of curved pills was significant (*MD* = -.093; *SD* = .23, *p* < .001).

The effect of benefit was also significant, *F* (2, 415) = 10.959, *p* < .001, partial *η*^*2*^ = .050. The energizing instruction led to significantly higher activations than calming instruction (*MD* = .120; *SE* = .026), *p* < .001. The neutral instruction led to significantly higher activations than calming instruction (*MD* = .069; *SE* = .025, *p* = .020).

The interaction between shape and benefit was significant, *F* (2, 415) = 3.057, *p* = .048, partial *η*^*2*^ = .015. The energizing effect of angularity was the highest when the pill’s proposed benefit was energizing, and vice versa, the calming effect of curvature enhanced when the pill’s proposed benefit was calming. The overall mean activation-deactivation values for angular and curved shapes (test value = 0) demonstrated that in energizing condition, the effects were only marginally significant for angular (*p* = .094) and curved (*p* = .074); in calming condition, the calming effect for curved pills (*p* < .001) and for angular pills (*p* < .001) were significant. In neutral condition, the energizing effect for angular pills was not significantly different from zero (*p* = .892), whereas calming effect of curved pills was significant (*p* < .001).

The effect of body part was significant, *F* (6.429, 2668.223) = 2.368, *p* = .026, partial *η*^*2*^ = .006, with Greenhouse-Geisser correction. There was a significant interaction between shape and body part, *F* (7.796, 3235.422) = 3.601, *p* = .001, partial *η*^*2*^ = .009. There was also a significant interaction between benefit and body part, *F* (12.859, 2668.223) = 3.791, *p* < .001, partial *η*^2^ = .018. There was no significant interaction between shape, benefit, and body part (*p* = .381) (see Fig 10 and S1 File for details).

#### Emotions associated with angular vs. curved pills

**Fig 11:**
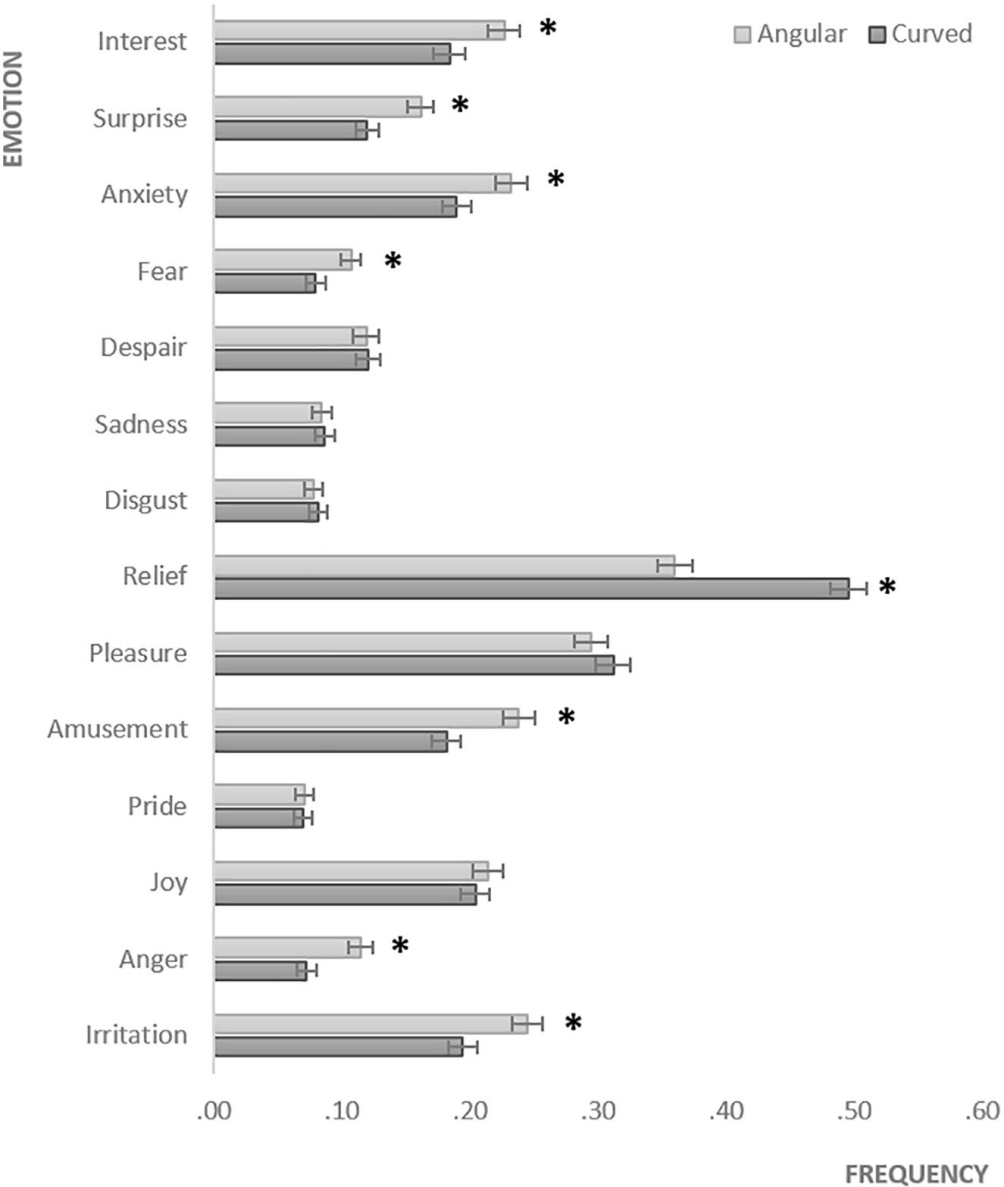
Angular vs. curved pills’ emotional associations. The bars represent SE. *Significant (p < .05) differences between angular and curved pills’ associations.

Paired-samples t-tests, conducted separately for each emotion, showed several significant differences between angular and curved pills’ associations (Fig 11). Mean frequency of interest (*p* = .001), surprise (*p* < .001), anxiety (*p* < .001), fear (*p* = .003), amusement (*p* < .001), anger (*p* < .001), irritation (*p* < .001) emotional associations was higher for angular than for curved pills, whereas mean frequency of relief (*p* < .001) associations was higher for curved pills.

A 2 X 3 Repeated Measures ANOVA (with Bonferroni correction) was conducted to assess the impact of within-subject shape (angular vs. curved), and between-subject benefit (energizing vs. calming vs. not-stated) variables on the number of emotional associations. The analysis revealed significant effect of shape, *F* (1, 415) = 1702.062, *p* < .001, partial *η*^2^ = .804, showing that pills’ angularity evoked more emotional associations than curvature (*MD* = 1.976; *SE* = .048). The effect of benefit (*p* = .155) and shape X benefit interaction (*p* = .172) were not significant.

### Discussion

The found effects of imagined ‘taking’ of curved vs. angular pills, presented as photograph stimuli, on bodily sensations further supported H1. In Study 3b, we found that angular shapes evoked overall more bodily activations than rounded shapes. However, as in Study 1, this difference was mostly due to calming effect of curvature. Furthermore, Study 3b showed that angular pills’ effects were not significant as compared to the baseline, but curved pills evoked significant increases in subjective calm feelings. In addition, the results of Study 3a demonstrated that angularity of a pill is associated with energizing effect, whereas curvature of a pill is associated with calming effect. Thus, overall, Study 3, provided additional support for H1. Notably, for pills-evoked changes in subjective feelings, the effect size was large for the shape and benefit effects but small for their interaction. For pills-evoked bodily sensations, all the effect sizes were rather small, but the effect size of shape was the largest among the others. The trends for bodily activations were consistent with the trends found for the reported energized vs. calm feelings.

In Study 3b, we also found that the instruction stating the proposed benefit of the pill (energizing vs. calming) lead to corresponding subjective effects (self-reported energized vs. calm feelings), thus supporting H2. The proposed energizing benefit yielded energizing effect for both angular and curved pills, whereas the proposed calming benefit yielded calming effect for both angular and curved pills. Moreover, when participants were informed about energizing vs. calming pills’ effects, their subjective ratings of energizing effects increased for angular pills, whereas their ratings of calming effects increased for curved pills as compared to neutral condition when participants were not informed about the pill’s benefits. Thus, we found support for the hypothesis that the congruity between the suggested benefit and the shape of the pill may improve the effectiveness of the pill (supporting H3). The patterns of bodily evoked activations vs. deactivations were generally consistent with the results from subjective ratings, however, as in Study 1 which also used pictorial stimuli, they were overall, shifted towards deactivated states. Finally, we found a number of significant differences between angular and curved pills’-evoked emotions (supporting H4). Overall, angular pills were associated with a greater number of emotions than curved pills, indicating that they evoked more blended emotional associations. In particular, angular pills were more frequently associated with interest, surprise, amusement knowledge emotions that are linked to the perception of novelty, unfamiliarity and complexity [46]. Also, angular shapes were more often than curved associated with negative emotions such as anxiety, fear, anger, and irritation. In contrast, a positive relief emotion was more frequently evoked by curved shapes than by angular shapes. Notably, the proposed pills’ benefits stated in the instructions did not significantly affect the number of emotional associations.

## General discussion

To our knowledge, this research is the first to find evidence that the shape of the pill has an impact on the perceived benefit of the drug, evoked subjective feelings and objective performance, bodily sensations, and emotional associations. The perceived effect is enhanced when the shape-induced expectations and the proposed benefit are congruent. These novel findings raise theoretical implications related to shape perception and effects of pills that come in different shapes. With some limitations, the findings also raise practical strategies and areas for future research.

In the current research, we found supporting evidence for H1 that the angular vs. curved shape of the pill has an effect on the perceived energizing vs. calming benefit of the pill, correspondingly. However, these effects depended on the nature of stimuli and instructions, and they were manifested differentially in the body. Across all studies, we consistently demonstrated that angular pills triggered more bodily activations compared to curvy pills. Though the overall effects of angularity vs. curvature on bodily sensations were generally consistent, some differences appeared. Pills’ angularity evoked more activations than curvature on a body level mostly due to calming effect of curvature in studies 1 and 3, but mostly due to energizing effect of angularity in Study 2. Such differences can be explained by the differences in stimuli: Studies 1 and 3 used drawings and photographs of the pills and were conducted via an online survey, whereas Study 2 employed realistic 3D mockups and required the imitation of swallowing act that probably triggered a higher level of activity. Possibly, using real pills would lead to even higher activations.

In Study 2, we found angularity of a pill not only evoked energized state feelings reflected in self-report ratings, but also led to the increased cognitive performance test scores, as compared to the baseline and as compared to the curvature-evoked self-report ratings and performance. Curved pills did not evoke significant calming subjective feelings or decreased performance test scores, as compared to the baseline. Thus, in Study 2 we only found evidence for energizing effects of angular pills but not calming effects of curved pills. Possibly, 3D pills led to energizing benefits and increased performance and also triggered more bodily activations since they are more realistic, and the participants actually simulated the physical act of swallowing. In Study 3a and 3b, we found evidence for both: energized effect of angular pills and calming effect of curved pills. Note, that in Study 3a, participants did not imagine ‘taking’ pills, but were directly asked about the possible effects of angular vs. curved pills. Overall, our study suggests that the shape of the pill itself (i.e., without any information about the proposed drug benefit) may communicate calming vs. energizing benefits. This finding is in line with past research that has documented that attributes extraneous to the drug such as color and size have an impact on the expectations about the pill benefits [7]. It is also consistent with some earlier studies showing that angularity is associated with more active states [39, 40, 47].

We also found that the proposed benefit of the pill resulted in corresponding subjective effects, supporting H2. That is, when individuals were instructed that they were ‘taking’ a pill with an energizing benefit, they reported higher levels of energy and when they were told that the pill had calming effects, they felt more calmed. Study 3b showed that energizing instruction led to significantly higher energized feelings than calming instruction; however, energizing instruction evoked only marginally significantly more energized feelings than neutral. The neutral instruction led to significantly higher energized feelings than calming instruction. We expect that study with 3D-printed or even real pills would reveal stronger effects of energizing instructions. Finally, in Study 3b we demonstrated that the effect of the pill was enhanced when the shape and the proposed benefit were congruent and attenuated when they were incongruent, supporting H3. The data from shape-evoked bodily sensations showed consistent trends. Our findings also converge with past research that maintains that designs in harmony with individual expectations are assessed as more effective [14]. Across the studies, the effect sizes for pills’ shape effects on the evoked bodily sensations, subjective feelings, performance and were overall the largest compared to other effects and interactions. The effect of proposed benefit also appeared to be large.

**Fig 12:**
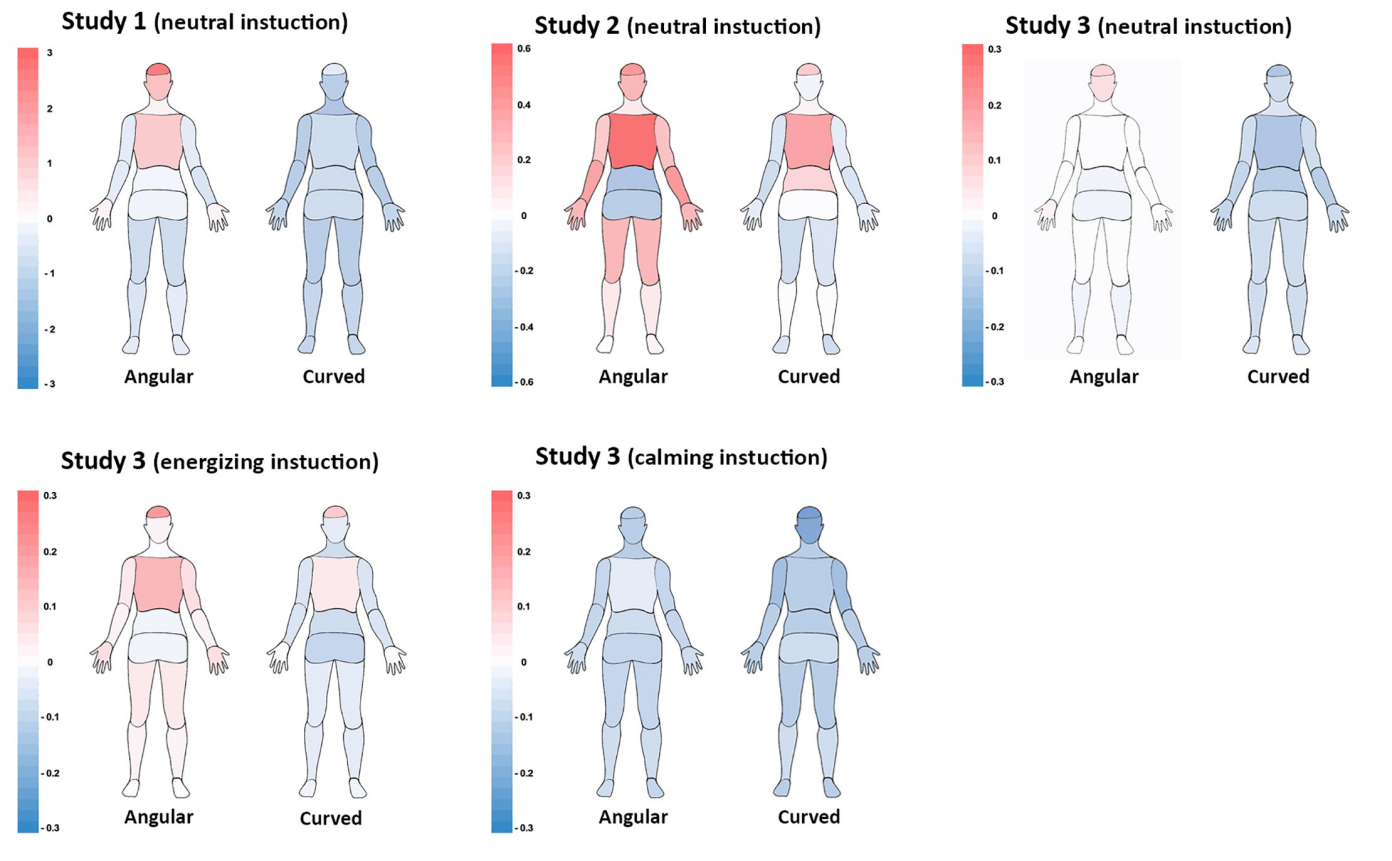
Topography of shape-evoked bodily activations-deactivations across the studies.

Additionally, we found that angular vs. curved pills evoked topographically distinct patterns of activations and deactivations in the body (Fig 12). Angular shapes triggered more activations than deactivations especially in the head (Studies 1, 2, 3-neutral), chest (Studies 1 and 2) and face (Studies 2 and 3-neutral) areas, as well as in limb (arms, hands and legs) areas (Study 2). Curved shapes evoked more deactivations in all body parts (Study 3-neutral) or in all areas except for the top of the head (Study 1). Across the studies, the most sensitive body areas affected by pills were head and chest areas. Similarly, Nummenmaa et al. [41] found head and chest areas to be significantly affected by different emotions. They reported that basic emotions yield elevated activity in the chest area, probably linked to changes in breathing and heart rate. Activations in the head area may denote physiological changes in the face (e.g., facial musculature activation, skin temperature) and refer to changes in the contents of mind. With the increased realism of the stimuli, we observed more activated bodily sensations in limbs. In particular, we found that angular 3D pills evoked activations in thighs, upper and lower arms as well as hands, possibly indicating readiness to perform an action. Similar, but smaller effects were observed in Study 3b (energizing condition) that used photographs of pills.

Interestingly, the pattern of activations and deactivations evoked by angular shapes appeared to be similar to those evoked by surprise emotion found in Nummenmaa et al. [41] study (activations in head and chest, deactivations in lower limbs). This is also consistent with our data on shapes’ emotional associations demonstrating that angularity of a pill is more frequently associated with interest, surprise, and amusement knowledge emotions [46]. Possibly, angular shapes are perceived as more unusual and interesting compared to round shapes [48]. Indeed, interest was shown to be more strongly related to physiological activation and appraisals of novelty [49, 46]. It is likely that angularity may affect higher cognitive processes and intellectual emotions leading to activations in head, whereas curvature may decrease cognitive control and mental processing. Furthermore, the additional activations in limbs evoked by angularity that were observed with increased realism of stimuli somewhat resembled the patterns of activations found in Nummenmaa et al. [41] for anger (activations in head, chest, upper limbs, and feet) and fear (activations in head, chest, abdomen, hands, and feet) emotions. Consistently, our data showed that angular pills more frequently than curved were associated with negative emotions such as anger, fear as well as irritation and anxiety. On the other hand, the pattern of activations evoked by curved pills in our study is somewhat similar to those evoked by depression as found in Nummenmaa et al. [41] study (deactivations in all body, especially in the head area). The current data showed that curved shape of the pill was more frequently associated with non-aroused and positive relief emotion (note, Nummenmaa et al. [41] did not have data for ‘relief’ emotion). Overall, the present study demonstrated the differences in shape-evoked emotions, supporting H4. Moreover, the data from emotional associations was generally consistent with data on shape-evoked bodily sensations.

The present research contributes to the existing literature in the fields of visual cognition, multisensory processing, and aesthetic perception. Specifically, it expands the research on angular vs. curved shape processing [24, 29, 37] and deepens the understanding of the angularity vs. curvature-evoked bodily sensations and emotional associations. Our results suggest that the perception of angular vs. curved shapes may trigger topographically distinct bodily sensations in terms of activations and deactivations.

The present findings have potential applications in various sectors such as marketing, pharmaceutical and dietary supplements industry, as well as other applied fields. Extant research in marketing shows that product design provides a clear advantage for the marketer alluring the consumer to the product [50–53]. Both current and previous research [22] suggest that curved vs. angular shapes used in marketing communications (e.g., packaging, logo) are linked to different needs and emotions, thus may trigger different expectations about the product and convey different benefits. As initial expectations about the drugs guide patient compliance [13], it is of utmost importance to amplify efficacy perceptions of pills. For example, medicine designed to restore mental alertness or wakefulness during fatigue (i.e., energizing) should be angular while a pill that is to cure sleep disorders (i.e., calming) should be round to maximize perceived effectiveness.

The current research holds promise for the use of these findings by the pharmaceutical industry, specifically the knowledge that angular and curved pills diversely affect different parts of the body. This finding could inform the shape design of the pills. The shape of the pill might be critical especially for those drugs that target certain body areas. For instance, the pill shape would matter when the pill is used for improving cognitive functions such as attention (i.e., angular pills activate the head).

We believe that these findings may also have implications beyond the pharmaceutical and dietary supplements industry. For example, the current findings can generalize to food perception; that is, the shape of the food may affect its perceived taste and health benefits. Our work opens several promising avenues for future research. First, further work is required to fully understand the underlying mechanisms of shape effects on perceived pills’ efficacy, bodily sensations and emotions. While we provide preliminary evidence in favor of a shape effect of pills on different bodily areas, emotions and perceived treatment effects, we did not test the potential mediating mechanisms (e.g., implicit associations of pills’ shapes) yielding these effects. Future research should look into underlying mechanisms of the suggested link.

Across three experiments, the stimuli varied in terms of ecological validity (abstract shape drawings in Study 1, 3D mock-ups in Study 2, and pictures of real pills in Study 3). However, none of our studies employed real pills. In all three experiments, we asked participants to imagine that they were ‘taking’ a pill. A large body of research suggests that mental imagery shares common neural and functional mechanisms with perception [54, 55], and it behaves as a weaker form of externally triggered perceptual experiences [56]. Research also showed that imagery-based situation models and mental simulations evoke sensorimotor representations [57, 58]. Thus, using an imagery task can provide an estimate for real pill taking. Nevertheless, a field research is warranted to see if our findings hold using real pills administered to users.

The current work uses angular and curvy pills. However, it should be borne in mind that the angular pills we employ are not truly angular (with clear sharp edges) which would potentially make the participants think that the pills would be difficult to swallow. Our manipulation checks nevertheless confirm that the angular pills were indeed perceived as angular. Future studies can systematically test how gradual deviations from shape angularity and curvature as well as their combinations may affect the pills’ perception. Notably, stimuli in Study 1 included two shape characteristics: shape outline and internal drawing. In our study they corresponded to each other; that is when the outline was angular, the internal drawing was also angular and vice versa. Future studies can test how the shape of the internal features (indentations or cut-outs) may affect the perception of the pill’s shape. One factor that may have affected our results is that participants may have already existing experiences of seeing/using pills that have shapes not matching their benefits. They may have already formed associations between certain shapes of pills and their benefits. Further development of 3D technology in pill design may overcome this potential problem as the designs will be novel to users.

Another area for future research is to test the proposed framework using different claimed benefits (e.g., relieving pain), different shapes and designs (e.g., indented), different colors and sizes of pills. In effect, rapidly developing 3D printing technology would allow for many opportunities that are yet unknown. Future pills may come in complex 3D shapes with additional features to maximize absorption rates (e.g., curved or angular notches or cut-outs). More research is needed to investigate these factors and their interactions on the perceived efficacy of the drug.

With this research, we examine an important, yet hitherto overlooked, question: What is the impact of shape of pills on expectancies, bodily sensations, emotions, and perceived effectiveness of the medicine? We believe that the current work can inform the pill design industry, providing a better framework to improve the effectiveness of the medicine. We hope that our findings inspire work toward a more comprehensive and nuanced account of the shape effects of pills.

## Supporting information

S1 File (DOCX)
