# Supplementary material for: The shape of the pill: Perceived effects, evoked bodily sensations and emotions
Source: PLoS One. 2020 Sep 8;15(9):e0238378. doi: 10.1371/journal.pone.0238378 (PMC7478620; doi:10.1371/journal.pone.0238378)
Supplement: S1 File — (DOCX) [file pone.0238378.s001.docx]

**Supplemental Materials**

**Study 1**

**Data cleaning**

Participants had to select between the options “I did the study with effort and I let my data to be used for research purposes” and “I answered randomly/ without effort. Don’t use my data.” (the latter was indicated by 5 participants). The data from those who claimed in the exit question that they answered randomly without effort, were removed.

**The effect of body part**

The mean activation-deactivation values were significantly higher for the top head (area #1) than all other areas (all p’s < .001 and; area #4, *p* = .032), and also they were higher for the chest area (area #4) than for areas #5 (*p* = .039), #6 (*p* = .084), #11 (*p* = .051), #12 (*p* = .047).

**The interaction between shape and body part**

For angular shapes, the activation-deactivation were significantly above the test value = 0 for body areas #1, #2, and #4 (all p’s ≤ .001), and they were significantly or marginally significantly below the test value = 0 for body areas #5 (*p* =.045), #6 (*p* =.084), #7 (*p* =.028), #8 (*p* =.012), #11, #12 (both *p*’s ≤ .001), #13 (*p* =.004) and #14 (*p* =.002). For curved shapes, the activation-deactivation were significantly below the test value = 0 for all body parts (all *p*’s ≤ .001, except for area #1, *p* =.280).

**Study 2**

**Identical Pictures performance test**


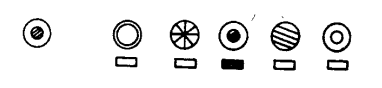
**S1 Fig. Sample item from Identical Pictures test.** Target figure is on the left; the correct response option, matching the target is marked.

**The effect of body part**

The mean activation-deactivation values were significantly higher for the chest (area #4) than in hip (area #10), *p* = .003.

**The interaction between shape and body part**

For angular shapes, the activation-deactivation were significantly above the test value = 0 for body areas #1 (*p* =.012), #2 (*p* =.002), #4 (*p* <.001), #6 (*p* =.030), #7 (*p* =.002), #8 (*p* <.001), #11 (*p* =.027), #12 (*p* =.025), #15 (*p* =.047), #16 (*p* =.038), but there were significantly below the test value = 0 for body areas #9 (*p* =.015) and #10 (*p* =.018). For curved shapes, the activation-deactivation were not significantly different from the test value = 0 for all body parts, except for area #4 which was above the test value (*p* =.015).

**The effect of time**

The evoked energetic *feelings* were significantly higher for after ‘taking’ than before ‘taking’ pills (*MD* = -563; *SE* = .192, *p* = .005). The *performance* was significantly higher for after ‘taking’ than before ‘taking’ pills (*MD* = .606; *SE* = .230, *p* = .010).

**The interaction between shape and time**

Pairwise comparisons showed that energetic *feeling* ratings after ‘taking’ angular pills were significantly higher than ratings at the baseline (*p* < .001). The ratings ‘after’ ‘taking’ curved pills were not significantly greater than ‘before’ ‘taking’ baseline ratings. Pairwise comparisons showed that *performance* scores for tests after ‘taking’ angular pills were significantly higher than scores at the baseline (*p* = .004). The scores after ‘taking’ curved pills were not significantly greater than the baseline scores.

**Study 3a**

**Data cleaning**

The data from participants who failed the attention check question (picking 'somewhat agree' option in a 7-point agree-disagree scale) were removed.

**Study 3b**

**Data cleaning**

First, participants, were asked to accurately mark hands and feet in specific colors on a body schema (failed by 88 participants). Second, participants were asked to indicate a specific number (5) on a 5-point scale (failed by 64 participants). Also, at the end of the survey, we asked participants to select between the options “I did the study with effort and I let my data to be used for research purposes” and “I answered randomly/ without effort. Don’t use my data.” (the latter was indicated by 15 participants). Among all the participants, there were 27 who failed both attention checks (5 of them also indicated that they answered randomly). Those who failed any attention check or claimed that they answered randomly without effort, were removed.

**The effect of body part**

Pairwise comparisons did not show significant differences.

**The interaction between shape and body part**

For angular shapes, the activation-deactivation were significantly above the test value = 0 for body area #1 (*p* =.031), but were significantly below the test value = 0 for body areas #3 (*p* =.015), #9 (*p* =.007), and #10 (*p* <. 001). For curved shapes, the activation-deactivation were significantly below the test value = 0 for all body parts (all *p*’s ≤ .001).

**Interaction between benefit and body part**

In energizing condition, the activation-deactivation were significantly above the test value = 0 for body areas #1 (*p* <.001) and #4 (*p* = .007) but were significantly below the test value = 0 for body areas #9 (*p* = .026) and #10 (*p* =. 012). In calming condition, the activation-deactivation were significantly below the test value = 0 for all body areas (*p* < .009). In neutral condition, the activation-deactivation were significantly below the test value = 0 for body areas #4 (*p* =.011), #5 (*p* =.028), #6 (*p* =.027), #7 (*p* = .003), #8 (*p* =.001), #9 (*p* = .001), #10 (*p* =.005), #13 (*p* = .008), #14 (*p* =.007), #17 (*p* = .045), #18 (*p* =.041).
